# A physics-based energy function allows the computational redesign of a PDZ domain

**DOI:** 10.1038/s41598-020-67972-w

**Published:** 2020-07-07

**Authors:** Vaitea Opuu, Young Joo Sun, Titus Hou, Nicolas Panel, Ernesto J. Fuentes, Thomas Simonson

**Affiliations:** 1 Laboratoire de Biologie Structurale de la Cellule (CNRS UMR7654), Ecole Polytechnique, Institut Polytechnique de Paris, Palaiseau, France; 2 Department of Biochemistry, Carver College of Medicine, University of Iowa, Iowa City, USA

**Keywords:** Biological models, Animal disease models

## Abstract

Computational protein design (CPD) can address the inverse folding problem, exploring a large space of sequences and selecting ones predicted to fold. CPD was used previously to redesign several proteins, employing a knowledge-based energy function for both the folded and unfolded states. We show that a PDZ domain can be entirely redesigned using a “physics-based” energy for the folded state and a knowledge-based energy for the unfolded state. Thousands of sequences were generated by Monte Carlo simulation. Three were chosen for experimental testing, based on their low energies and several empirical criteria. All three could be overexpressed and had native-like circular dichroism spectra and 1D-NMR spectra typical of folded structures. Two had upshifted thermal denaturation curves when a peptide ligand was present, indicating binding and suggesting folding to a correct, PDZ structure. Evidently, the physical principles that govern folded proteins, with a dash of empirical post-filtering, can allow successful whole-protein redesign.

## Introduction

Protein sequences have been selected by evolution to fold into specific structures, stabilized by a subtle balance of interactions involving protein and solvent^1,2^. In contrast, random polymers of amino acids are very unlikely to adopt a specific, folded structure^3,4^, and exhibit instead a more disordered structure^5^. A powerful approach to understand the evolution of proteins and the basis of folding is to perform computer simulations that mimic evolution. This can be done with computational protein design (CPD), which explores a large set of sequences and selects ones predicted to adopt a given fold^6–8^. A typical simulation imposes a specific geometry for the protein backbone, corresponding to the experimental conformation of a natural protein. Side chains are mutated randomly. Variants with a favorable predicted folding free energy are saved. The folded state energy function can be physics-based or knowledge-based^9–11^ while the unfolded state energy is knowledge-based. The protein is considered “redesigned” if most of the protein side chains are allowed to mutate during the simulation.

The successful redesign of complete proteins was reported in 2003^7,12^ and small miniproteins were redesigned even earlier^6,13^. Several other successes were obtained^14–17^, including a study where 15000 miniproteins (40–43 amino acids) were redesigned^18^. 6% of the designs were shown to be successful; i.e., the designed miniproteins folded into the correct tertiary structure. The others either could not be overexpressed and purified, or did not fold as predicted. All of the applications to proteins described the folded structure with an energy function that was at least partly knowledge-based, or statistical. Statistical energy terms included terms derived from experimental amino acid propensities and evolutionary covariances^17^, terms derived from inter-residue distance distributions in crystal structures^16^, and terms derived from torsion angle and hydrogen-bond distance distributions in crystal structures^11,14,15^. All of the applications described the unfolded structure with a fully statistical, knowledge-based model.

Energy functions for the folded state can also be non-empirical, or physics-based, and taken from molecular mechanics^19^. There are then only two energy terms for nonbonded interactions between protein atoms, which correspond to the elementary Coulomb and Lennard-Jones effects. Their parameterization relies mainly on fitting quantum chemical calculations performed on small model compounds in the gas phase. The solvent is described implicitly, using varying levels of approximation^20^. The most rigorous model used so far is a dielectric continuum model^21^. This requires solving a differential equation, which is technically impractical in a protein design framework. Therefore, a Generalized Born (GB) approximation is more common. GB contains much of the same physics but provides a simpler, analytical energy expression^20^. GB models have been studied extensively in the context of protein design but also molecular dynamics, free energy simulations, acid/base calculations, ligand binding and protein folding^22–25^. They reproduce the behavior of the dielectric continuum model rather accurately. Therefore, an energy function that combines molecular mechanics for the protein with a GB solvent can be considered “physics-based”, even though it is not entirely constructed from first principles. A molecular mechanics energy, combined with a very simple solvent model, was used to design two artificial proteins that each consisted of a four-helix bundle, where an elementary unit of 34 amino acids was replicated four times^26,27^. However, until now, there has not been a complete, experimentally-verified redesign of a natural protein using a physics-based energy function for the folded protein.

Here, we report the successful use of a physics-based energy function to completely redesign a PDZ domain of 83 amino acids. PDZ domains (“Postsynaptic density-95/Discs large/Zonula occludens-1”) are globular domains that establish protein-protein interaction networks^28^. They interact specifically with target proteins, usually by recognizing a few amino acids at the target C-terminus. They have been extensively studied and used to elucidate principles of protein evolution and folding^29,30^. Our design started from the PDZ domain of the Calcium/calmodulin-dependent serine kinase (CASK) protein. It used the backbone conformation from a new, high-resolution X-ray structure of apo CASK reported here. Several other CASK X-ray structures are also known, with bound peptides. The CASK melting temperature is about 10 °C higher than that of the Tiam1 PDZ domain, which we attempted to redesign earlier^33^. This increased thermostability could aid in retrieving folded CASK designs. Design was performed by running long Monte Carlo (MC) simulations where most positions were allowed to mutate and all positions could explore a library or conformers, or rotamers. Positions occupied by glycine (seven) or proline (two) were not allowed to mutate. 13 positions that directly contact a peptide ligand in CASK:peptide complex structures (such as PDB 6NID) also kept their wild-type identity. All 61 of the other side chains (73.5% of the sequence) were allowed to mutate freely into any amino acid type except Gly or Pro, for a total of 3.7 × 10^76^ possible sequences. To describe the folded state, we used a physics-based energy function that combined the Amber molecular mechanics force field^31^ and a GB solvent^32^. To describe the unfolded state, we used a knowledge-based energy function^33^. The Proteus software was used^34^. Three sampled sequences, or designs were chosen for experimental testing, based on their low energies and several empirical criteria. All three were shown to fold, with good evidence the folded structure was the target, native PDZ fold. In particular, secondary structure content was native-like and binding to one or two peptides that are known CASK ligands was demonstrated for two of the three designs. Therefore, the redesign is considered a success. Evidently, the physical principles that govern folded proteins, as captured by molecular mechanics and continuum electrostatics are sufficient to allow whole-protein design, at least when assisted by a moderate empirical post-filtering. This is encouraging, since these methods give physical insights, can be systematically improved, and are transferable to nucleic acids, sugars, noncanonical amino acids, and ligands of biotechnological interest.

## Results

**Figure 1:**
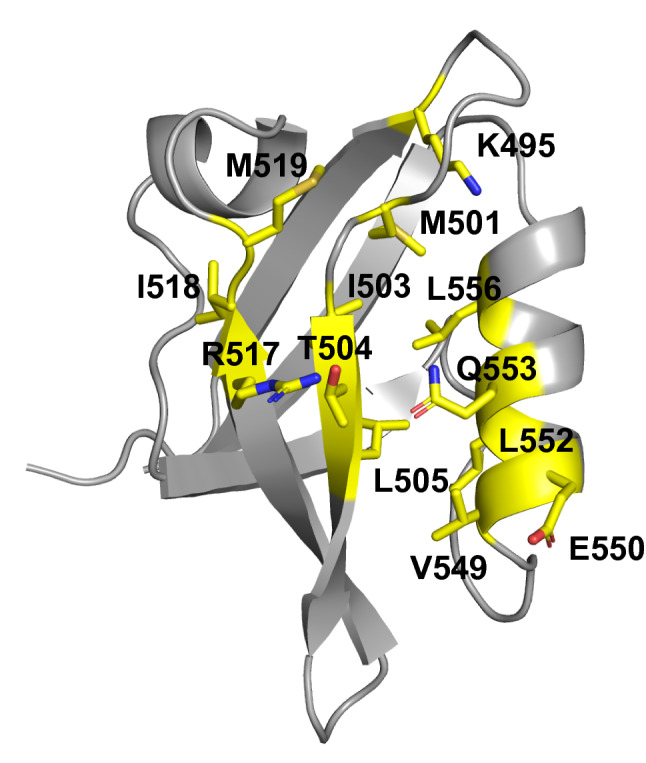
CASK 3D structure. The 13 amino acids in yellow are involved in ligand binding and were not allowed to mutate in the simulations.

MC simulations were done using the CASK backbone conformation (Fig. 1). The method is detailed in Supplementary Material. 61 of 83 residues were allowed to mutate into all types except Gly and Pro. 13 residues known to be directly involved in peptide binding were not allowed to mutate (but could explore rotamers). The exploration did not use any bias towards natural sequences or any limit on the number of mutations. The 2,000 sequences with the lowest folding energies were kept for analysis. Below, we describe their computational characterization and the selection of three representative sequences for experimental characterization.

### Computational characterization and sequence selection

**Figure 2:**
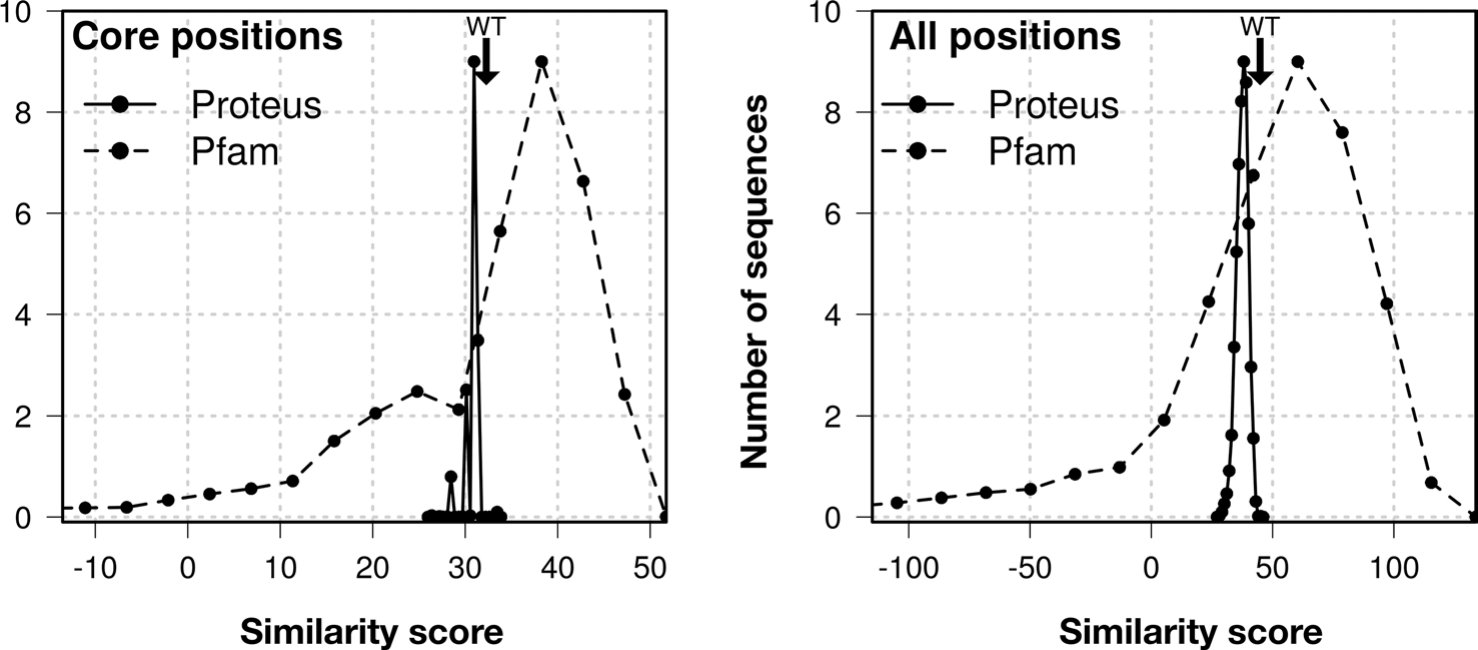
Blosum similarity scores compared to natural Pfam sequences. Black line: histogram of scores for the top 2,000 Proteus sequences, considering only 15 core positions (left) or all positions (right). Dashed line: scores for the Pfam sequences themselves. WT CASK score is indicated by an arrow.

The top 2,000 sequences spanned a folding energy interval of 1.5 kcal/mol. They were analyzed by the Superfamily fold recognition tool^35^, which assigns sequences to SCOP^36^ structural families. None of the top 2,000 Proteus sequences were assigned by Superfamily to any other fold in SCOP; all were recognized as belonging to the PDZ family. Blosum40 similarity scores between the designed sequences and natural sequences from the Pfam database were also computed (Fig. 2). The scores were high, and comparable to those of natural PDZ domains. The peaks in the Proteus histograms are narrow, indicating that the 2,000 lowest-energy sequences are similar to each other. Similarities to CASK are in Supp. Material (Fig. S1).

To narrow down the number of sequences for experimental testing, we excluded those with isoelectric points estimated to be close to the physiological pH, between 6.5 and 8.5, which might be subject to aggregation and difficult to express. This reduced the number of sequences from 2,000 to 1,268. Next, we used a criterion of negative design, by considering the confidence levels for the Superfamily assignments to the PDZ family, instead of another SCOP family. Of the 1,268 sequences left, we only retained those that had Superfamily match lengths above the mean value (over the 1,268) and E-values above the mean (log$_{10}$ E < − 31). This left us with 692 sequences. We reduced the number further using four empirical criteria. (1) We excluded sequences with similarity scores versus Pfam below the mean (over the 692 remaining sequences). This eliminated a window of candidate sequences about 10 points wide, to the left of the mean, plus a few sequences in the lefthand tail of the distribution. We were left with 215 sequences. (2) We excluded sequences that had a cavity buried in the predicted 3D structure. (3) We required a total unsigned protein charge of less than 6. (4) We allowed no more than 15 mutations that drastically changed the amino acid type (defined by a Blosum62 similarity score between the two amino acid types of − 2 or less).

**Figure 3:**
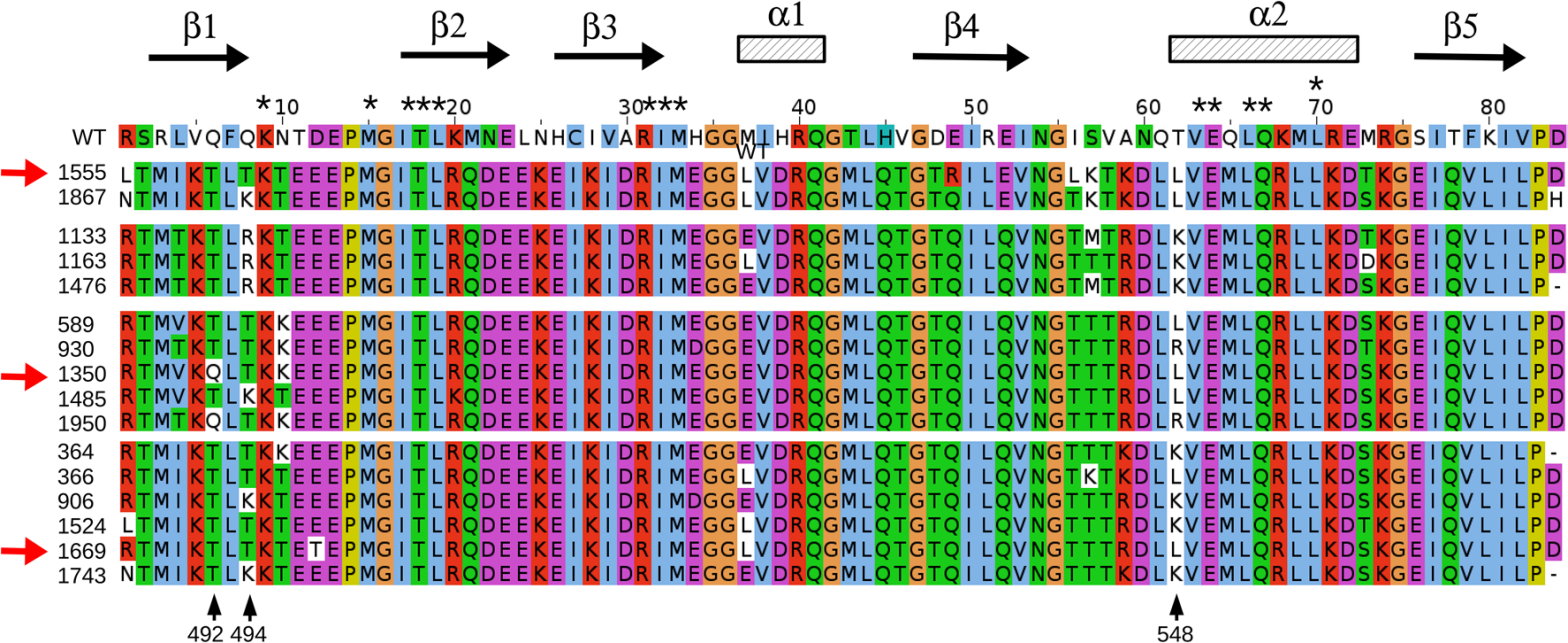
WT and the 16 final candidate designed sequences based on the CASK template (Clustal colors). The sequences tested experimentally are indicated by red arrows. Asterisks (above) indicate positions not allowed to mutate during the design, in addition to Gly, Pro.

We were left with 16 candidate sequences, shown in Fig. 3. They were separated into four groups by visual inspection. Group 2 was eliminated based on its Arg494 residue, absent from CASK homologs. One candidate was selected from each of the other groups (highlighted in Fig. 3), with a preference for native or homologous residue types at positions 492 (candidate 1350), 494 (candidate 1555), and 548 (candidate 1669)—positions that are close to the peptide binding interface. The three candidates were simulated by molecular dynamics with explicit solvent for one microsecond each, and their stabilities and flexibilities appeared comparable to the wild-type (Supplementary Material, Figs. S2–S3). Therefore, the three sequences were retained for experimental testing. The number of mutations, compared to wild-type CASK, were 50 (candidate 1350), and 51 (candidates 1555 and 1669), representing just over 60% of the sequence.

### Experimental characterization of selected sequences

#### Earlier designs based on the Tiam1 template

Computational redesign of Tiam1 was described earlier^33^. It used the Tiam1 PDZ domain structure (PDB code 4GVD; Supplementary Material, Fig. S4). The GB electrostatics model included an additional “Native Environment Approximation” (NEA)^37^, where each atom experienced a constant dielectric environment that corresponded to the native sequence and conformation (see Computational Methods in Supplementary Material). This removed the many-body character of the GB model and made the calculations very efficient. Eight designs were expressed and purified. Their yields were low. CD gave spectra typical of random coil polymers, suggesting the proteins were misfolded (Supplementary Material, Fig. S5). 1D-NMR spectra of the amide region of the NEA designs had limited dispersion and broad resonances compared to the native Tiam1 PDZ domain, corroborating the CD data. An example is shown below; others are in Fig. S6. Differential scanning fluorimetry (DSF) in the presence of known Tiam1 ligands did not show any binding by the Tiam1 NEA designs, while the Tiam1 PDZ domain showed robust binding (Supplementary Material, Fig. S7). Together, these data indicate that the NEA-based designs of the Tiam1 PDZ domain could be overexpressed but adopted unfolded structures, unable to bind known Tiam1 peptide ligands.

#### Designs based on the CASK template

**Figure 4:**
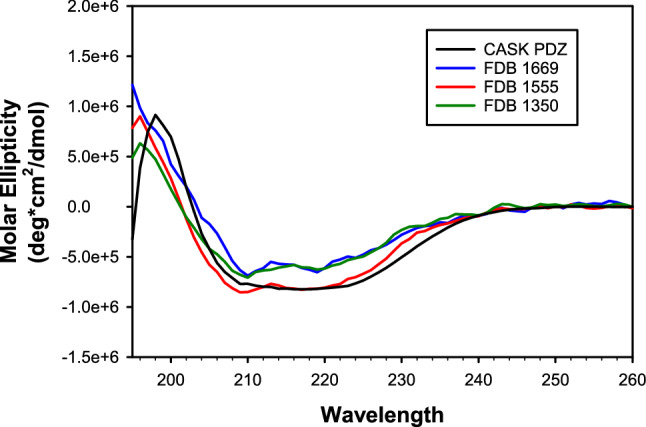
Circular dichroism spectra of a natural PDZ domain (CASK, black) and three selected designs based on the CASK template and the FDB electrostatic model. FDB-1350 (green), FDB-1555 (red), and FDB-1669 (blue) all have $\alpha$ helix and $\beta$ strand signals similar to a native PDZ domain like CASK (black). The concentration of each protein ranged from 10 to 20 μM.

**Figure 5:**
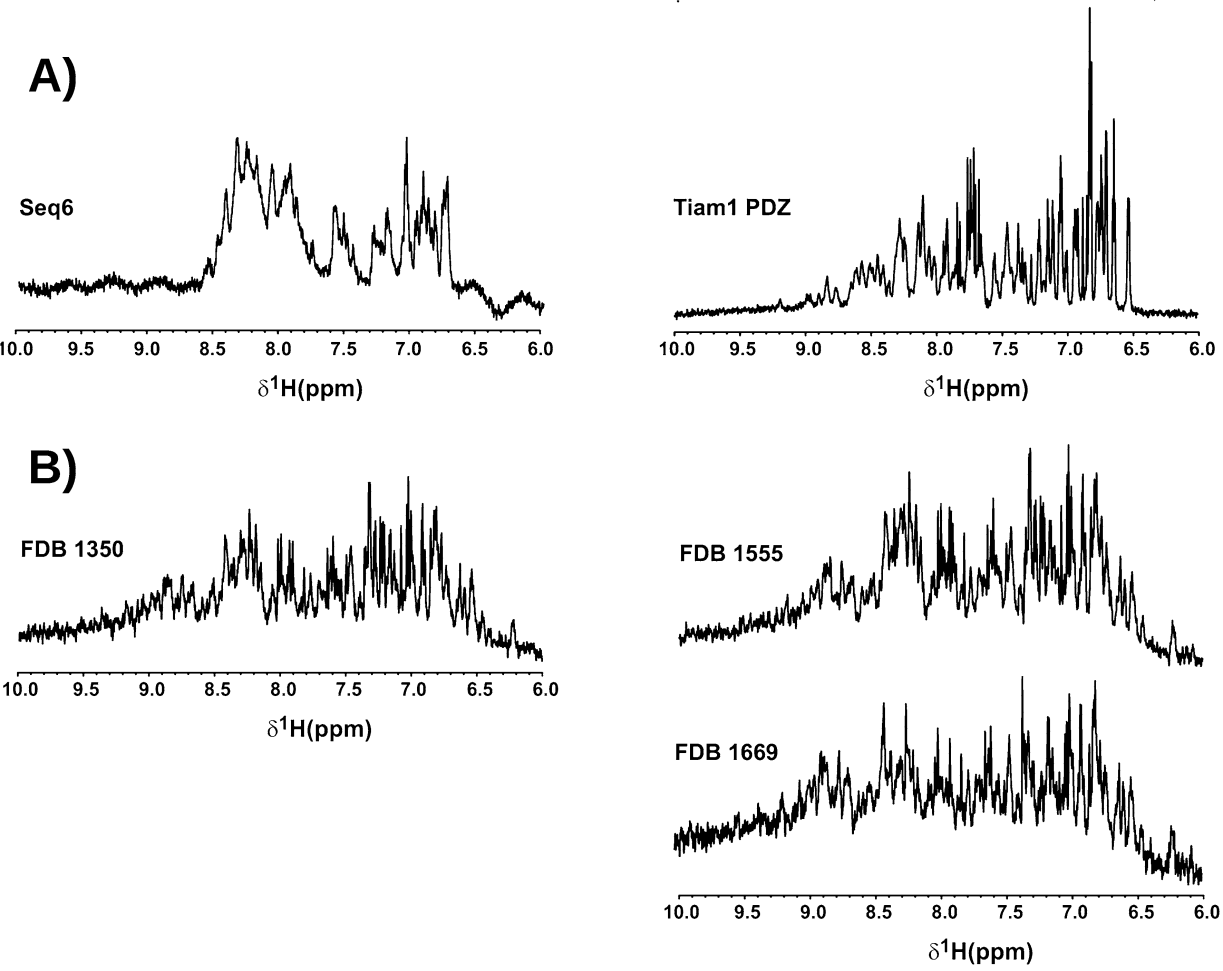
Proton NMR spectra of the natural Tiam1 PDZ domain and selected designs. (**A**) Left: a design obtained with the Tiam1 template and the NEA electrostatic model; right: Tiam1. (**B**) 3 designs obtained based on the CASK template and the FDB electrostatic model. The concentration of the designed proteins ranged from 14 to 22 μM; Tiam1 concentration was 150 μM.

Next, we characterized the three designs selected above, which we refer to as FDB-1350, FDB-1555, and FDB-1669. They were obtained using as template a new apo CASK PDZ domain structure (PDB code 6NH9, reported here). The Tiam1 and CASK backbone conformations have a small rms deviation of 1.0 Å, despite a low sequence identity of 20.5%. CASK has a ~ 10 °C higher melting temperature, which could facilitate its redesign. The new calculations used a more rigorous GB electrostatics model (Supplementary Material), termed the “Fluctuating Dielectric Boundary” model (FDB)^38^. With this model, the dielectric environment of each atom was updated on-the-fly during the simulation, instead of being represented by a mean environment. The expression yields in *E. coli* were improved over the NEA Tiam1 designs, though not to the level typically seen with native PDZ domains. In contrast to the NEA Tiam1 designs, CD spectra were similar to native PDZ domains, suggesting these designs were structured (Fig. 4). 1D-proton NMR of the amide region showed good dispersion and sharp lines, consistent with a folded protein (Fig. 5B) and in contrast to the earlier, Tiam1 redesigns (Figs. 5A and S6). The designed proteins’ spectra had noisier baselines, due to a seven- to tenfold lower concentration, compared to CASK.

**Figure 6:**
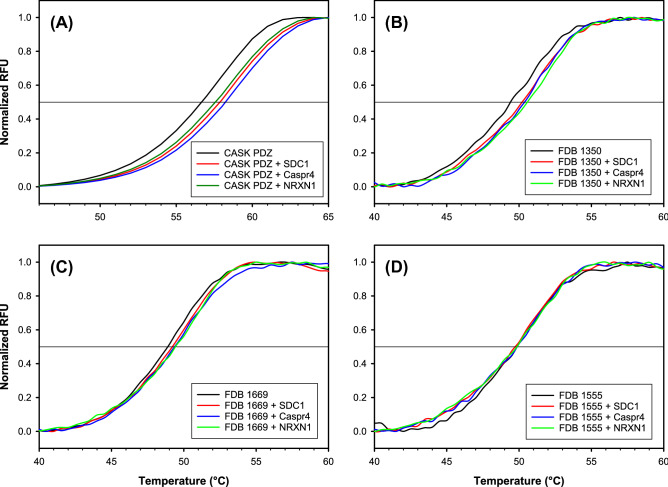
Differential scanning fluorimetry of (**A**) a natural PDZ domain (CASK) and (**B**–**D**) three selected designs based on the CASK template and the FDB electrostatic model. Signals in the absence and presence of the SDC1, Capr4 and NRXN peptides.

We tested the ability of the designs to bind CASK ligands, using DSF experiments. The CASK PDZ domain showed binding to SDC1, Caspr4 and NRXN (Fig 6 and Table 1), as expected. Strikingly, two of the three CASK FDB designs characterized also showed binding to some of the peptides. Thus, FDB-1350 had a significant thermal shift in the presence of NRXN and SDC1. FDB-1669 showed a 1.0 °C change in T$_{1/2}$ in the presence of the NRXN peptide. In contrast, FDB-1555 did not show significant thermal shifts in the presence of any peptide. From these data, we conclude that the three CASK FDB designs were folded and two were capable of interacting with peptide ligands. In principle, the CD and NMR spectra could be obtained with an alternative protein fold, distinct from the target PDZ fold. However, the structural data clearly indicate that the designs are well-ordered and have a secondary structure content similar to the CASK target. Importantly, the ordered character, the secondary structure content, the ability to bind CASK ligands, the structural stability during microsecond MD runs, and the Superfamily classification as a PDZ domain strongly suggest that the designed proteins adopt the target PDZ fold.

**Table 1:** DSF for wild-type CASK and three Proteus designs.

Protein^a^	T$_{1/2}$ ($^{\circ }$C) and $\delta$T$_{1/2}$ = T$_{1/2}^{\mathrm{apo}}$ − T$_{1/2}$ (in parentheses)				Binding^b^
	Apo	SDC1	Caspr4	NRXN	
CASK PDZ	57.2 ± 0.2	58.4 ± 0.1	58.7 ± 0.1	58.1 ± 0.2	**SDC1, Caspr4**
		(+ 1.2)	(+ 1.5)	(+ 0.9)	NRXN
FDB-1350	49.8 ± 0.4	50.7 ± 0.2	50.4 ± 0.4	51.3 ± 0.2	SDC1
		(+ 0.9)	(+ 0.6)	(+ 1.5)	**NRXN**
FDB-1669	49.1 ± 0.1	49.6 ± 0.1	49.5 ± 0.0	50.1 ± 0.1	**NRXN**
		(+ 0.4)	(+ 0.4)	(+ 1.0)	
FDB-1555	49.9 ± 0.2	50.2 ± 0.1	50.3 ± 0.1	50.5 ± 0.6	–
		(+ 0.3)	(+ 0.5)	(+ 0.6)	

^a^Protein concentration was ~ 25 μM (about 0.25 mg/ml). Peptide concentration was 300 μM.

^b^When $\delta$T$_{1/2}$ was larger than sum of the standard deviation of apo and each peptide, we considered the peptides to have a significant change in T$_{1/2}$, indicating binding to the PDZ domain. ± indicates standard deviation of three biological replicates.

Peptides in bold (right column) produced the largest changes.

## Discussion

Protein folding is thought to be induced by protein–solvent and solvent–solvent interactions^39^, since folding buries nonpolar groups and allows waters to interact with polar amino acid side chains and other waters. In this picture, the protein dielectric properties play a role, with the low-dielectric interior pushing polar protein groups out towards high-dielectric solvent. The protein nonpolar surface also plays a role, with exposed surface leading to fewer water–water interactions^40^. Thus, it is common to discuss protein solvation in terms of nonpolar and electrostatic components, and most implicit solvent models rely on this separation^20^. Small proteins have been found to fold correctly in MD simulations with both explicit solvent and accurate implicit solvent models^22,41^, which can all be considered physics-based. The inverse folding problem is even more complex, since it explores an enormous space of sequences, albeit with a reduced conformation set. Modeling the solvent is a key step to solve this problem, and a key ingredient of our procedure.

The first solvation component in our model is nonpolar and uses accessible surface areas and atomic surface tensions. Nonpolar solvation of a large collection of small molecules correlated well with surface area^42^, supporting this treatment. The surface tension parametrization was updated recently, compared to our earlier Tiam1 designs^43^. Surface interactions in proteins are complex and have a many-body character^6,32^, since three or more groups can have surfaces that all overlap. Our model explicitly includes backbone-side chain triple overlaps, while others are accounted for implicitly^43^.

The largest solvation effects are electrostatic, and they also have a many-body character. Indeed, a side chain can desolvate an interacting pair, affecting the strength of their interaction. The electrostatic, Generalized Born component of our model captures this effect. However, for previous Tiam1 design calculations^33^, we had used an approximation where each protein residue experienced a constant, native-like, dielectric environment. This removed the many-body character of electrostatic solvation. The Tiam1 designs were shown here to be largely unsuccessful: the proteins could be overexpressed but were only weakly structured. In contrast, preserving the many-body solvation was shown previously to give improved accuracy for side chain pK$_a$’s^38^ and increased similarity between CPD sequences and natural sequences of several PDZ proteins^38^. Therefore, for the CASK redesign, we applied the newer, many-body FDB model and obtained improved results. We did not test whether the improved, FDB model would have also produced valid designs with the Tiam1 backbone as the template.

Our calculations used a CASK X-ray structure reported here, determined at 1.85 Å resolution. In our design procedure, the protein backbone was held fixed in the X-ray conformation, while side chains mutated and explored rotamers. More precisely, the backbone motions were not ignored but were treated implicitly, through the protein dielectric constant, $\epsilon _P$. The value used here, $\epsilon _P=4$, is known to be physically reasonable for proteins. MD simulations further showed that the tested sequences have backbone structures very similar to the wild-type protein and native-like flexibilities.

While our folded state model was physics-based, the design procedure included two other elements that were knowledge-based. For the unfolded state, we assumed a simple, extended peptide model, to which an empirical correction was added that involved type-dependent amino acid chemical potentials^37^. All successful whole-protein redesigns have used similar, knowledge-based unfolded models. Second, we used several filters to choose a handful of sequences for experimental testing, and most of the filters were empirical. Indeed, the folded and unfolded models are imperfect, and while they produced at least three sequences that fold correctly (true positives), they presumably also produced false positives. The empirical filtering did not affect the sequence design, but was used to increase the chances that we would pick true positives for experimental testing. Starting from sequences within 1.5 kcal/mol of the top folding energy, we used the (computed) isoelectric point to reduce the chances of aggregation. We also used negative design, based on the Superfamily fold recognition tool. Indeed, negative design against aggregation or alternate folds was not included in the MC calculations. This left us with 692 designed sequences. Next, we eliminated sequences whose Blosum similarity to natural PDZ sequences was below the average of the 692 remaining sequences. This criterion was not very stringent, because the distribution of the Blosum scores was already very narrow (see Fig. 2, right panel, solid line and Fig. S1). At this point, we were left with 215 sequences. We then eliminated sequences whose structural models included large cavities and ones with a large net charge, which could lead to electrostatic repulsion within the folded structure. Finally, we eliminated sequences with more than 15 “drastic” mutations (corresponding to Blosum scores of − 2 or less). This left us with 16 sequences. We chose 3 that were representative.

The three tested proteins could be overexpressed, had sharp 1D-NMR peaks typical of a folded protein and native-like CD spectra. Two exhibited a shift of their thermal denaturation in the presence of one or two peptides that are known CASK ligands. Evidently, our physics-based folded model and empirical unfolded model can be used to successfully redesign a whole protein, at least with the help of some empirical post-filtering. The expression yields, protein solubilities and stabilities of the designed sequences were lower than for wild-type CASK, so that it was not possible to produce large amounts of pure protein for 2D-NMR or X-ray crystallography. It may be possible to improve this behavior by testing a larger number of designs, by using a more sophisticated filtering of candidate sequences for solubility (beyond estimating the isoelectric point), or by improving the physical model even further. Model improvements might include backbone-dependent rotamers and/or multiple backbone conformations.

The present design method, which combines molecular mechanics, continuum electrostatics, and Boltzmann sampling, is an example of physics-based CPD. It is striking and encouraging that this approach allows whole protein redesign to be done successfully. We expect that the physics-based route will increasingly yield valuable insights and should be a valuable complement to knowledge-based CPD and experimental design.

## Supplementary information

Supplementary material 1
