# Supplementary material for: A physics-based energy function allows the computational redesign of a PDZ domain
Source: Sci Rep. 2020 Jul 7;10:11150. doi: 10.1038/s41598-020-67972-w (PMC7341745; doi:10.1038/s41598-020-67972-w)
Supplement: Supplementary file 1 — Supplementary material 1 [file 41598_2020_67972_MOESM1_ESM.pdf]

Supplementary Material: A physics-based energy function allows the computational redesign of a PDZ domain

Vaitea Opuu[a,†], Young Joo Sun[b,†], Titus Hou[b], Nicolas Panel[a], Ernesto J. Fuentes[b,*] & Thomas Simonson[a,*]

[a]Laboratoire de Biochimie (CNRS UMR7654), Ecole Polytechnique, Palaiseau, France
[b]Dept. of Biochemistry, Carver College of Medicine, University of Iowa, Iowa City, USA

Below, we first provide Material and Methods. Then, we provide supplementary Results. We report sequence similarities between the Proteus designed sequences and the CASK sequence. We provide information on the stability and flexibility of the CASK-based designs in microsecond molecular dynamics (MD) simulations. We report the experimental characterization of PDZ sequences designed using the Tiam1 template structure and the NEA electrostatics model. Finally, we report the crystallographic structure statistics for the apo CASK PDZ domain.

Table of contents

1. Materials and Methods S2
1.1 Computational design methods S2
1.2 Protein expression and purification S6
1.3 Crystal structure of the wild-type apo CASK PDZ domain S7
1.4 Biophysical characterization of designed proteins S7
2 Supplementary Results S9
2.1 Sequence similarities between designed sequences and CASK S9
2.2 Stability of the three selected CASK-based designs in molecular dynamics S10
2.3 Experimental characterization of Proteus designs obtained with the Tiam1 template and the NEA electrostatic model S12
2.4 Human apo CASK PDZ domain X-ray structure statistics S15

1 Material and Methods

1.1 Computational design methods

Energy function for the folded state

We used the following energy function for the folded state:

$$E = E_{\mathrm{MM}} + E_{\mathrm{GB}} + E_{\mathrm{SA}} \tag{1}$$

E_{MM} is the protein internal energy, taken from the Amber ff99SB molecular mechanics (MM) energy function [1]. E_{GB} is a Generalized Born (GB) implicit solvent contribution [2, 3]:

$$E_{\mathrm{GB}} = \frac{1}{2}\left(\frac{1}{\epsilon_W} - \frac{1}{\epsilon_P}\right)\sum_{ij} q_i q_j \left(r_{ij}^2 + b_i b_j \exp[-r_{ij}^2/4b_i b_j]\right)^{-1/2} \tag{2}$$

Here, ϵ_W and ϵ_P are the solvent and protein dielectric constants (80 and 4, respectively); r_{ij} is the distance between atoms i, j and b_i is the "solvation radius" of atom i [2, 4]. The dependency of the b_i on the protein conformation corresponds to a GB variant we call GB/HCT (for "Hawkins-Cramer-Truhlar") [2, 4]. For some of the design calculations, an additional "Native Environment Approximation", or NEA was used for efficiency [3, 5], where the solvation radius b_i of each particular group (backbone, sidechain or ligand) was computed ahead of time, with the rest of the system having its native sequence and conformation [6, 7]. For the other designs, we computed the solvation radii on the fly during the MC simulation, using a very fast implementation called "Fluctuating Dielectric Boundary," or FDB [7] that uses lookup tables.

The last term in Eq. (1) is a surface area term:

$$E_{\mathrm{SA}} = \sum_i \sigma_i A_i \tag{3}$$

A_i is the exposed solvent accessible surface area of atom i; σ_i is a parameter that reflects each atom's preference to be exposed or hidden from solvent. The solute atoms were divided into four groups with specific σ_i values. The values were -60 (nonpolar), 30 (aromatic), -120 (polar), and -110 (ionic) cal/mol/Å^2. The coefficient for hydrogens was zero. Negative values are physically correct, since the SA term includes favorable protein-solvent dispersion interactions, in addition to hydrophobic effects. Surface areas were computed by the Lee and Richards algorithm [8], implemented in the Proteus software [5], using a 1.5 Å probe radius. Surface burial is not additive, since the same area on a

side chain can be buried by two other residues. To avoid overcounting, a scaling factor was applied to the contact areas involving at least one buried side chain [9]. In previous tests, a value of 0.65 gave the best surface areas, compared to an exact calculation [3, 4].

The unfolded state energy

For a sequence S, the unfolded energy is:

$$E^u = \sum_{i \in S} E^u(t_i, B_i). \tag{4}$$

The sum is over all amino acids; t_i represents the side chain type at position i; B_i represents the buried or exposed character of position i in the folded state. The quantities $E^u(t, B) \equiv E^u_t$ an be thought of as effective chemical potentials of each amino acid type. Their values were chosen empirically, to maximize the likelihood of a set of experimental PDZ sequences. This means that an MC simulation should give overall amino acid frequencies that match those in the experimental sequences [10]. We assigned different values to buried and exposed positions, because we assume residual structure is present in the unfolded state, so that amino acids partly retain their buried/exposed character. Thus, the simulations should reproduce the overall composition of the buried and exposed positions separately. To define the target amino acid frequencies for likelihood maximization, we used a set of PDZ sequences collected earlier [10]. CASK positions were considered buried or exposed based on their solvent-accessible surface area in the CASK 3D structure, with a threshold designed to place roughly half of the positions in either category. Positions in the other PDZ sequences were considered buried or exposed based on a sequence alignment that included CASK: positions aligned with a buried CASK position were buried. Likelihood maximization was initiated with $E^u(t, B)$ values obtained from a non-empirical, tripeptide model [10, 11]. The first iterations then optimized the frequencies of 11 groups of homologous amino acid types [10]. This corresponds to 20 independent, adjustable, unfolded energies (10×2 independent groups). The values after convergence (16 iterations) are reported in Table S1. They differ only moderately from the initial, non-empirical values. 5 more iterations were done to optimize the individual type frequencies. This corresponds to 34 adjustable unfolded energies (17 independent types, since Gly and Pro were not allowed, × 2 regions) [10]. In these iterations, the energies changed very little, by 0.15 kcal/mol on average. Thus, while the number of parameters is large, the departure from the non-empirical values is very small.

Table S1: Unfolded energies (kcal/mol)

	Exposed positions			Buried positions		
	initial[a]	interm.[b]	final[c]	initial[a]	interm.[b]	final[c]
ALA	0.00	0.00	0.00	0.00	0.00	0.00
ARG	-54.76	-56.54	-56.85	-51.37	-51.85	-52.00
ASN	-17.80	-20.01	-20.13	-14.02	-14.34	-14.44
ASP	-18.82	-19.95	-20.11	-14.55	-14.57	-14.76
CYS	-1.64	-1.64	-1.78	-1.06	-1.06	-1.01
GLN	-16.61	-18.82	-19.25	-13.14	-13.46	-13.53
GLU	-18.21	-19.34	-19.80	-14.52	-14.54	-14.52
HIS_δ	7.37	6.94	6.66	10.41	10.57	10.54
HIS_ϵ	8.12	7.69	7.41	10.85	11.01	10.98
HIS^+	10.98	10.55	10.27	12.86	13.02	12.99
ILE	3.06	2.48	2.41	5.50	5.40	5.43
LEU	-2.94	-3.52	-4.03	0.00	-0.10	-0.09
LYS	-11.35	-10.69	-10.88	-8.24	-7.70	-7.65
MET	-3.09	-3.94	-4.26	-2.85	-2.09	-1.90
PHE	-3.18	-3.27	-3.32	0.17	0.77	0.93
SER	-5.24	-5.23	-5.46	-4.45	-4.24	-4.26
THR	-6.68	-6.68	-7.09	-4.84	-4.84	-4.96
TRP	-5.53	-5.62	-5.74	-1.94	-1.34	-1.30
TYR	-10.14	-10.29	-10.36	-5.91	-5.56	-5.50
VAL	-1.66	-2.24	-2.25	-0.05	-0.15	-0.30

[a]Initial values from tripeptide model. [b]Optimized for 11 groups of amino acid types (20 independent parameters). [c]Optimized for each amino acid type, which corresponds to 34 independent, adjustable parameters. With respect to the 20-parameter stage, the mean E^u change was just 0.15 kcal/mol.

Structural model and energy matrix

For CASK, we used a new X-ray structure of the apo PDZ domain, reported here (PDB entry 6NH9). To carry out the MC simulations, an energy matrix was computed using procedures described previously [10]. Briefly, for each pair of amino acid side chains, the interaction energy was computed after 15 steps of energy minimization, with the backbone held fixed and only the interactions of the pair with each other and the backbone included [12]. Side chain rotamers were described by the Tuffery library [13], expanded to include additional hydrogen orientations for OH and SH groups [3]. The energies were stored in an energy table, or "matrix" for use during MC.

Monte Carlo simulations

Sequence design was performed by running long MC simulations where 61 out of 83 positions could mutate freely: all but 7 Gly, 2 Pro and 13 positions that are directly involved in binding the peptide ligand. Non-mutating positions could explore different rotamers. The MC simulations used one- and two-position moves, where either rotamers, amino acid types, or both changed. For two-position moves, the second position was near the first in space. Sampling was enhanced by using Replica Exchange Monte Carlo (REMC), where eight MC simulations ("replicas") were run in parallel, at different temperatures [14]. Periodic swaps were attempted between the conformations of two replicas i, j (adjacent in temperature), subject to a Metropolis acceptance test [14]. Thermal energies ranged from 0.125 to 3 kcal/mol. Simulations were done with the Proteus software [5, 14].

Molecular dynamics simulations

Wild-Type CASK and six sequences designed with Proteus were subjected to MD simulations with explicit solvent and no peptide ligand. The starting structures were taken from the MC trajectory or the crystal structure and slightly minimized with harmonic restraints to maintain the backbone geometry. Each protein was immersed in a solvent box using the CHARMM GUI [15, 16]. The boxes had a truncated octahedral shape. The minimum distance between protein atoms and the box edge was 15 Å. The final models included about 11,000 water molecules. A few sodium or chloride ions were included to ensure overall electroneutrality. The protonation states of histidines were assigned to be neutral, based on visual inspection. MD was performed with periodic boundary conditions, at room temperature and pressure, using Langevin dynamics with a Langevin

Piston Nosé-Hoover barostat [17, 18]. Long-range electrostatic interactions were treated with a Particle Mesh Ewald approach [19]. The Amber ff14SB force field and the TIP3P model [20] were used for the protein and water, respectively. Simulations were run for one microsecond, using the Charmm and NAMD programs [16, 21].

1.2 Protein expression and purification

The codon optimized gene of the human CASK PDZ domain (residues 487–572) was chemically synthesized (GenScript Inc., Piscataway, NJ) and ligated into the pET28a vector (Novagen). The DNA sequence of the pET28a-CASK PDZ vector was verified by automated DNA sequencing (University of Iowa, DNA Facility). Protein expression was conducted in BL21(DE3) (Invitrogen) *E. coli* cells. Typically, *E. coli* cells were grown at 37°C in Luria-Bertani (LB) medium supplemented with kanamycin (15 μg/mL) under vigorous agitation until an absorbance at 600 nm wavelength (A600) reached 0.6-0.8. Cultures were subsequently cooled to 18°C and protein expression was induced by the addition of isopropyl 1-thio-β-d-galactopyranoside (IPTG) to 1 mM final concentration. Induced cells were incubated for an additional 16–18 hrs at 18°C. and harvested by centrifugation. The CASK PDZ domain was purified by cation exchange (SP media, GE-Healthcare) and size-exclusion chromatography (GE-Healthcare). Superdex 75 (S75) size-exclusion chromatography was performed with desired final buffer (20 mM phosphate, pH 6.8, 50 mM NaCl, and 0.5 mM EDTA). The final yield was 50 mg of CASK PDZ protein >98% pure as judged by SDS-PAGE from 1 L of culture. Samples were used immediately or lyophilized and stored at -80°C. The Tiam1 PDZ domain was purified as previously published [22].

The genes encoding the Proteus PDZ designs were codon-optimized for *E. coli* expression and chemically synthesized by GenScript Inc. (Piscataway, NJ). The genes were cloned into a modified pET21a vector (Novagen) that contains a His_6-tag and Tobacco etch virus protease cleavage site at the 5′-end of the multiple cloning site. The nucleotide coding sequence of the pET21a-PDZ vector was verified by automated DNA sequencing (University of Iowa, DNA Facility). Protein expression was conducted in BL21(DE3) (Invitrogen) *E. coli cells.* Typically, cells were grown at 37°C in Luria-Bertani medium supplemented with ampicillin (100 μg/mL) under vigorous agitation until an A600 of 0.6–0.8 was reached. Cultures were subsequently cooled to 18°C and protein expression was induced by the addition of IPTG to 1 mM final concentration. Induced cells were incubated for an additional 16-18 hrs at 18°C and harvested by centrifugation. Proteins

were initially purified by nickel-chelate chromatography (GE-Healthcare). The proteins were further purified by size-exclusion chromatography (Superdex 75, GE Healthcare) using a buffer containing 20 mM phosphate, pH 6.8, 50 mM NaCl, and 0.5 mM EDTA. Samples were used immediately.

1.3 Crystal structure of the wild-type apo CASK PDZ domain

A crystal structure of the apo CASK PDZ domain was determined in this work. High-throughput hanging-drop, vapor-diffusion screens using a Mosquito drop setter (TTP LabTech) were used to determine the crystallization conditions. The CASK PDZ domain was prepared in 20 mM Tris pH 7.5 and 50 mM NaCl. 200 nL of precipitant and PDZ domain (10-30 mg/mL) was used for each screening condition. Initial screening for diffracting crystals was done with an in-house Rigaku RAXIS-IV rotating anode X-ray source. Collection of full X-ray diffraction datasets for structure determination was done at beamline 4.2.2 at the Advanced Light Source (Berkeley, CA). Proper space group handedness was verified by analysis of the electron density.

XDS was used for indexing, integration, and scaling of the diffraction data [23, 24], to 1.85 Å resolution. XSCALE was used to merge multiple datasets. We used PHASER and previously-determined PDZ structures for initial phasing [25]. We used Refmac [26, 27] for the early stages of refinement and PHENIX [28, 29] for the final refinement. Refinement statistics are given in Supplementary Information (Table S1). Manual model building was done based on visualized electron density in Coot [30, 30]. 4.6% of the reflections were randomly selected to be excluded from the refinement and used to calculate R_{free} values. Alignment of structures and generation of figures were done with PyMOL (Schrodinger, LLC, The PyMOL Molecular Graphics System).

1.4 Biophysical characterization of designed proteins

Synthetic peptides

All peptides were chemically synthesized by GenScript Inc. (Piscataway, NJ) and were >95% pure as judged by analytical HPLC and mass spectrometry. Peptides were dansylated at the N-terminus and had a free carboxyl at the C-terminus. The peptides used in this study were derived from the following proteins: Neurexin (residues 1,470–1,477: NKDKEYYV_{COOH}), Caspr4 (residues 1,301–1,308: ENQKEYFF_{COOH}) and Syndecan1

(residues 303–310: TKQEEFYA$_{COOH}$).

Circular dichroism

Circular dichroism signals were measured using a Jasco J-815 circular dichroism spectropolarimeter. The concentration of each protein ranged from 10 to 20 μM. All proteins were in a buffer composed of 20 mM phosphate, pH 6.8, 50 mM NaCl, and 0.5 mM EDTA. Spectra were taken from the 190 nm to 260 nm wavelength window with a 1 nm data interval at 25°C. Data integration time was 2 seconds and the scanning speed was 100 nm/min.

NMR

Nuclear magnetic resonance (NMR) experiments were carried out at 298 K (calibrated with methanol) on Bruker Avance II 800 MHz (equipped with a CryoProbe), Bruker Avance II 500 MHz, and Varian 600 MHz spectrometers (equipped with room temperature probes). All protein samples were prepared in 20 mM phosphate, pH 6.8, 50 mM NaCl, 0.5 mM EDTA, and 10% (v/v) D_2O with a concentration of 14 μM to 22 μM.

Differential scanning fluorimetry

Standard methodology was used for differential scanning fluorimetry (DSF) [31, 32]. Briefly, DSF was performed using 96-well PCR plates and the Sypro Orange (Thermo Fisher) dye. Each well in the PCR plate had a 20 μL final volume containing 0.25 mg/mL of protein, 300 μM of peptide, and 5x Sypro Orange final concentration (from a 5000x stock) in a buffer containing 20 mM phosphate, pH 6.8, 50 mM NaCl, and 0.5 mM EDTA. The DSF assays were performed using a Bio-Rad CFX96 real-time polymerase chain reaction instrument equipped to read 96-well plates. The protein of interest was thermally denatured from 5°C to 95°C at a ramp rate of 1°C/min. The protein melting/unfolding curves were generated by monitoring changes in Sypro Orange fluorescence (at 610 nm wavelength). Raw fluorescence data were analyzed using DMAN, and the first derivative value from the denaturation data was used to determine the apparent melting temperature [33] ($T_{1/2}$). Each peptide was assayed in triplicate. A 96-well plate containing no peptide was assayed to determine the apparent $T_{1/2}$ of each PDZ domain in the absence of any peptide. A shift of more than 1°C in $T_{1/2}$ indicates binding (based on SEM).

2 Supplementary Results

2.1 Sequence similarities between designed sequences and CASK

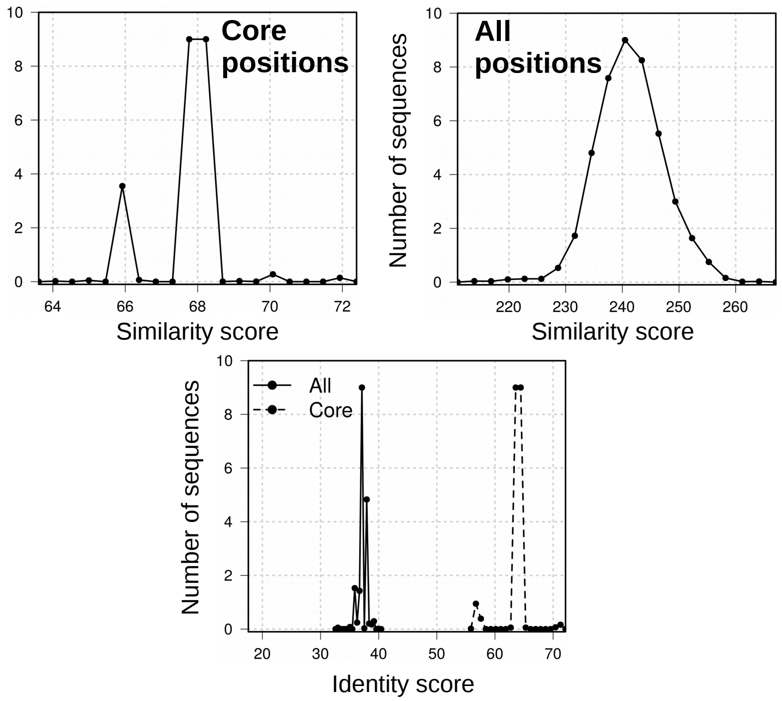

Figure S1: Histograms of Blosum40 similarity scores (above) and sequence identities (below) compared to CASK, for the 2000 lowest-energy designed sequences.

2.2 Stability of the three selected CASK-based designs in molecular dynamics

As a first test of the three selected sequences, FDB1350, FDB1555, and FDB1669, they were subjected to MD simulations using an explicit solvent environment, for 1000 ns. Wild-Type CASK (WT) was also simulated. Convergence of the simulations was good (based on a principal component analysis, not shown). The WT protein was quite stable, with rms deviations from the starting, X-ray structure of 1–1.5 Å (excluding 3–4 residues at each terminus and one very flexible loop, residues 495–502; see Fig. S2). Deviations from its own mean MD structure were similar (Fig. S2). The designed proteins exhibited only slightly larger deviations from the WT X-ray structure (1.2–1.8 Å) and similar, small deviations from their respective mean MD structures, with no visible drift (Fig. S2).

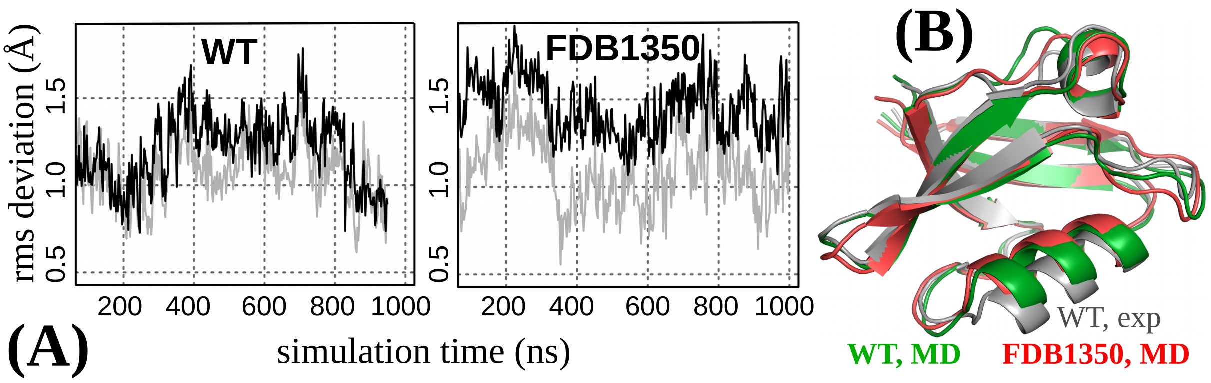

Figure S2: MD simulations of CASK-based designs. **A)** Backbone rms deviations for WT and the FDB1350 designed variant relative to the starting structure (black) and the mean MD structure (grey). **B)** Mean MD structures of WT and designed variant FDB1350.

We also characterized the backbone flexibility of the designed proteins by computing NMR order parameters for the backbone amide groups (Fig. S3). Experimental values were not available for WT CASK, but were available for Tiam1 and a quadruple mutant of Tiam1 [34]. These proteins were also simulated by MD for one microsecond, with and without the peptide ligands Sdc1 and Caspr4, respectively. In Fig. S3, we show the order parameters for both proteins in the apo and holo states, from experiment (circles) and MD (continuous lines) (top two panels). The agreement is very good. Next, we show (Fig. S3, bottom panel) the order parameters for WT CASK and the three selected CASK-based designs, FDB1350, FDB1555, and FDB1669 (apo proteins). Comparing the designed

proteins to WT CASK, the results were similar, with some differences in loop regions. Two designs were slightly less flexible than WT (see positions 492-502 in β_1-β_2, 521-524 in β_3-α_1), while FDB1350 was slightly more flexible (see 492–496 in β_1-β_2 and 559-561 in α_2-β_5). Evidently, the design calculations do not produce overly-rigid or overly-flexible proteins in a systematic way.

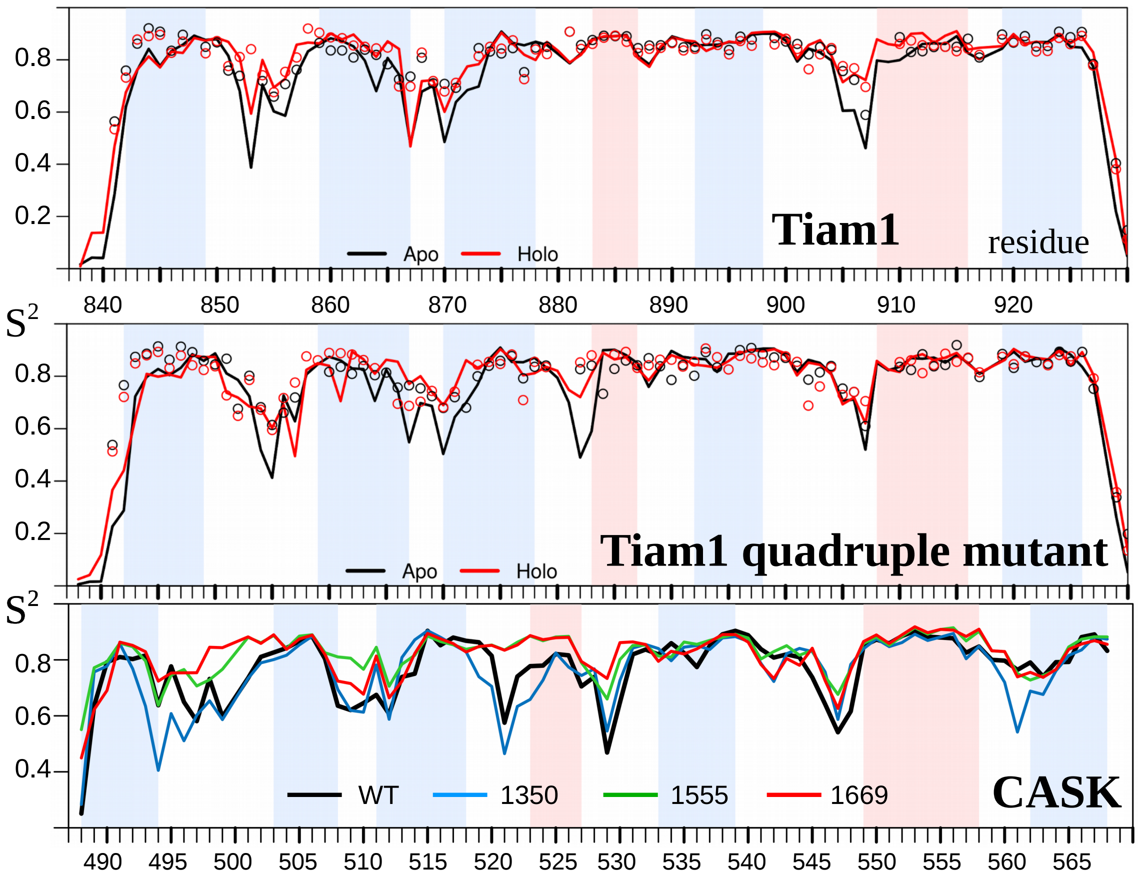

Figure S3: Backbone amide NMR order parameters for natural and designed proteins. **Top panel:** Tiam1 with and without the Sdc1 peptide ligand. Circles are experimental values; lines are from μsec MD simulations. **Middle panel:** analogous data for the Tiam1 quadruple mutant and the Caspr4 peptide. **Bottom panel:** Apo WT CASK and the three designed variants; values from MD.

2.3 Experimental characterization of Proteus designs obtained with the Tiam1 template and the NEA electrostatic model

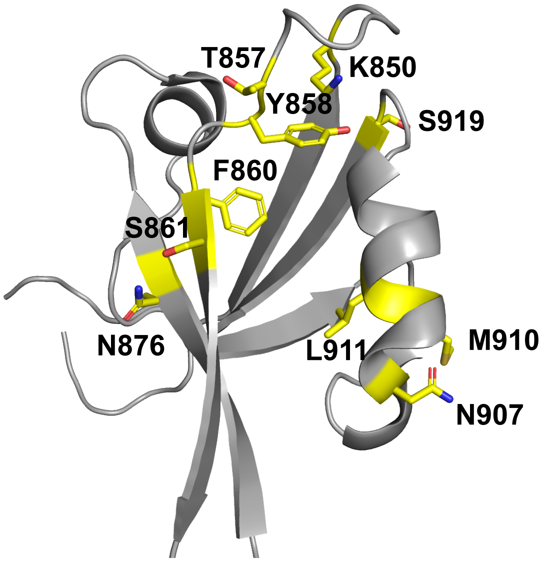

Figure S4: Tiam1 structure. Yellow: 13 positions whose types were fixed in the Proteus designs.

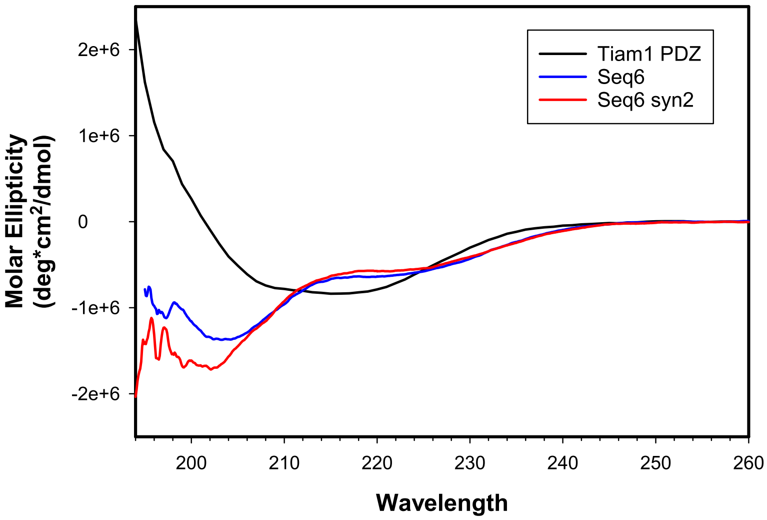

Figure S5: CD spectra of Tiam1 and two designs based on the Tiam1 template and NEA electrostatics.

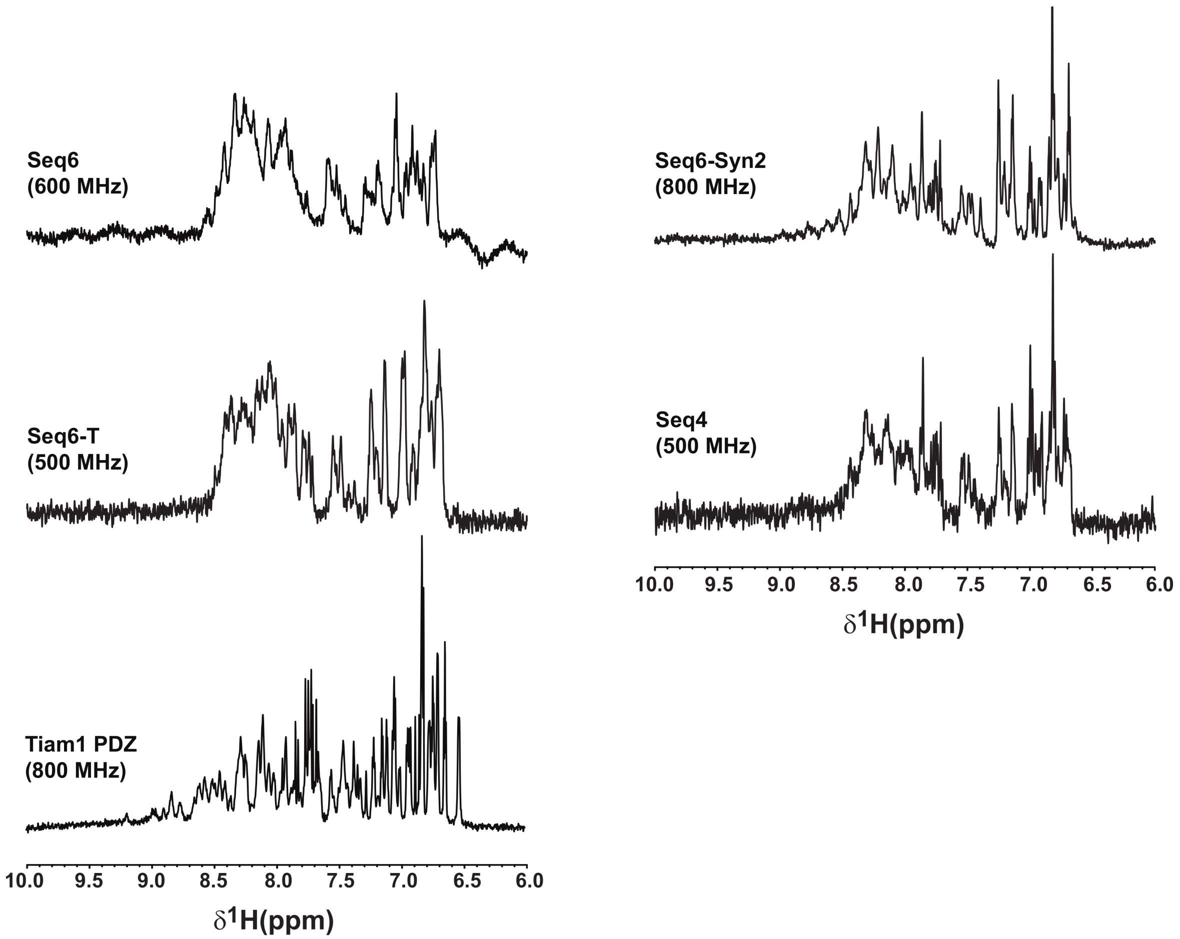

Figure S6: Proton NMR spectra of the Tiam1 PDZ domain and four designs obtained with the Tiam1 template and NEA electrostatics.

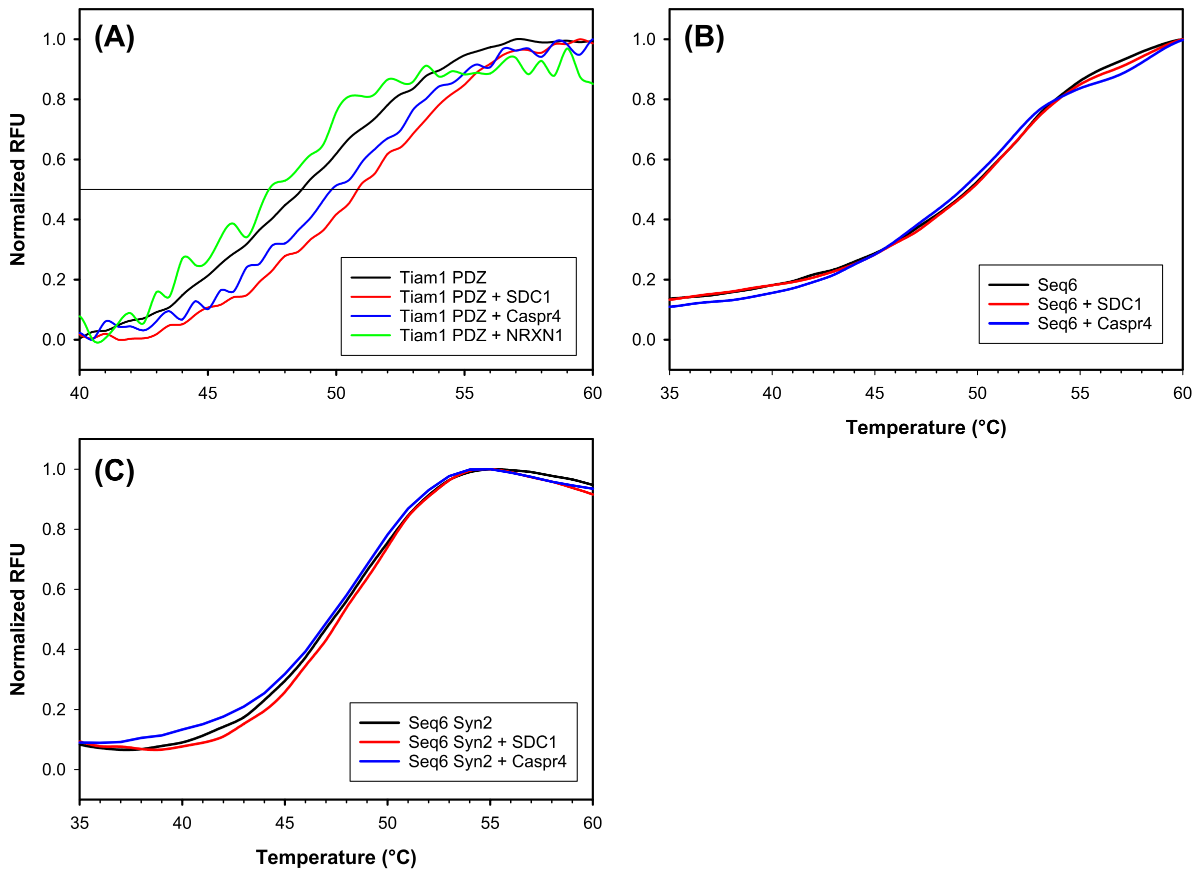

Figure S7: Differential scanning fluorimetry of a natural PDZ domain (Tiam1) and two designs based on the Tiam1 template and the NEA electrostatic model. Signals in the absence and presence of the SDC1, Capr4 and NRXN peptides.

2.4 Human apo CASK PDZ domain X-ray structure statistics

Table S2: Crystallographic statistics for the human apo CASK PDZ domain

Data collection statistics	
Beam line	ALS 4.2.2
Wavelength (Å)	1.0003
Space group	C 1 2 1
Unit cell dimensions (a, b, c) (Å)	61.1, 35.4, 119.5
Unit cell dimensions (α, β, γ)	90°, 90.3°, 90°
Resolution range (Å)	59.8—1.85
Total reflections	37,385 (7,461)
Unique reflections	20,769 (1,910)
Multiplicity	1.8 (1.7)
Completeness (%)	93.7 (93.7)
I/σ (I)	10.4 (2.1)
Wilson B-factor (Å^2)	50.7
Rmeas	0.030 (0.402)
$CC_{1/2}$	99.8 (91.1)
**Refinement statistics**	
Resolution (Å)	1.85
No. of reflections used in refinement	20,739 (2,705)
No. of reflections used for R_{free}	964 (133)
R_{work}/R_{free}	0.226/0.263
No. of atoms (Protein/Water)	4,188 (4,037/151)
B-factors (Å^2)	53.0
R.M.S.D.[a]	
Bond length (Å)	0.29
Bond angle (degrees)	0.46
Ramachandran plot statistics (%)	
In preferred regions	98.0
In allowed regions	2.0
Outliers	0.0
PDB accession code	6NH9

Numbers in parentheses are for the highest-resolution shell. [a]RMS deviation from ideal values.

References

[1] Cornell, W. *et al.* A second generation force field for the simulation of proteins, nucleic acids, and organic molecules. *J. Am. Chem. Soc.* **117**, 5179–5197 (1995).

[2] Hawkins, G. D., Cramer, C. & Truhlar, D. Pairwise descreening of solute charges from a dielectric medium. *Chem. Phys. Lett.* **246**, 122–129 (1995).

[3] Gaillard, T. & Simonson, T. Pairwise decomposition of an MMGBSA energy function for computational protein design. *J. Comput. Chem.* **35**, 1371–1387 (2014).

[4] Lopes, A., Aleksandrov, A., Bathelt, C., Archontis, G. & Simonson, T. Computational sidechain placement and protein mutagenesis with implicit solvent models. *Proteins* **67**, 853–867 (2007).

[5] Simonson, T. *et al.* Computational protein design: the Proteus software and selected applications. *J. Comput. Chem.* **34**, 2472–2484 (2013).

[6] Simonson, T. Protein:ligand recognition: simple models for electrostatic effects. *Curr. Pharma. Design* **19**, 4241–4256 (2013).

[7] Villa, F., Mignon, D., Polydorides, S. & Simonson, T. Comparing pairwise-additive and many-body generalized born models for acid/base calculations and protein design. *J. Comput. Chem.* **38**, 2396–2410 (2017).

[8] Lee, B. & Richards, F. The interpretation of protein structures: estimation of static accessibility. *J. Mol. Biol.* **55**, 379–400 (1971).

[9] Dahiyat, B. I. & Mayo, S. L. De novo protein design: fully automated sequence selection. *Science* **278**, 82–87 (1997).

[10] Mignon, D., Panel, N., Chen, X., Fuentes, E. J. & Simonson, T. Computational design of the Tiam1 PDZ domain and its ligand binding. *J. Chem. Theory Comput.* **13**, 2271–2289 (2017).

[11] Pokala, N. & Handel, T. M. Energy functions for protein design: adjustment with protein-protein complex affinities, models for the unfolded state, and negative design of solubility and specificity. *J. Mol. Biol.* **347**, 203–227 (2005).

[12] Schmidt am Busch, M., Lopes, A., Mignon, D. & Simonson, T. Computational protein design: software implementation, parameter optimization, and performance of a simple model. *J. Comput. Chem.* **29**, 1092–1102 (2008).

[13] Tuffery, P., Etchebest, C., Hazout, S. & Lavery, R. A new approach to the rapid determination of protein side chain conformations. *J. Biomol. Struct. Dyn.* **8**, 1267–1289 (1991).

[14] Mignon, D. & Simonson, T. Comparing three stochastic search algorithms for computational protein design: Monte Carlo, Replica Exchange Monte Carlo, and a multistart, steepest-descent heuristic. *J. Comput. Chem.* **37**, 1781–1793 (2016).

[15] Jo, S., Kim, T., Iyer, V. G. & Im, W. CHARMM-GUI: a web-based graphical user interface for CHARMM. *J. Comput. Chem.* **29**, 1859–1865 (2008).

[16] Brooks, B. *et al.* CHARMM: The biomolecular simulation program. *J. Comput. Chem.* **30**, 1545–1614 (2009).

[17] Martyna, G. J., Tobias, D. J. & Klein, M. L. Constant pressure molecular dynamics algorithms. *J. Chem. Phys.* **101**, 4177–4189 (1994).

[18] Feller, S. E., Zhang, Y., Pastor, R. W. & Brooks, B. R. Constant pressure molecular dynamics simulation: the Langevin piston method. *J. Chem. Phys.* **103**, 4613–4622 (1995).

[19] Darden, T. Treatment of long-range forces and potential. In Becker, O., MacKerrell Jr., A. D., Roux, B. & Watanabe, M. (eds.) *Computational Biochemistry & Biophysics*, chap. 4 (Marcel Dekker, N.Y., 2001).

[20] Jorgensen, W. L., Chandrasekar, J., Madura, J., Impey, R. & Klein, M. Comparison of simple potential functions for simulating liquid water. *J. Chem. Phys.* **79**, 926–935 (1983).

[21] Phillips, J. C. *et al.* Scalable molecular dynamics with NAMD. *J. Comput. Chem.* **26**, 1781–1802 (2005).

[22] Shepherd, T. R. *et al.* The Tiam1 PDZ domain couples to Syndecan1 and promotes cell-matrix adhesion. *J. Mol. Biol.* **398**, 730–746 (2010).

[23] Kabsch, W. Integration, scaling, space-group assignment and post-refinement. *Acta Cryst. D* **66**, 133–144 (2010).

[24] Kabsch, W. XDS. *Acta Cryst. D* **66**, 125–132 (2010).

[25] McCoy, A. J. *et al.* Phaser crystallographic software. *J. Appl. Cryst.* **40**, 658–674 (2007).

[26] Murshudov, G. N., Vagin, A. A. & Dodson, E. J. Refinement of macromolecular structures by the maximum likelihood method. *Acta Cryst.* **D53**, 240–255 (1997).

[27] Vagin, A. A. *et al.* REFMAC5 dictionary: organization of prior chemical knowledge and guidelines for its use. *Acta Cryst. D* **60**, 2184–2195 (2004).

[28] Adams, P. D. *et al.* PHENIX: building new software for automated crystallographic structure determination. *Acta Cryst. D* **58**, 1948–1954 (2002).

[29] Adams, P. D. *et al.* PHENIX: a comprehensive Python-based system for macromolecular structure solution. *Acta Cryst. D* **66**, 213–221 (2010).

[30] Emsley, P., Lohkamp, B., Scott, W. G. & Cowtan, K. Features and development of Coot. *Acta Cryst. D* **66**, 486–501 (2010).

[31] Ehrhardt, M. K. G., Warring, S. L. & Gerth, M. L. Screening chemoreceptor-ligand interactions by high-throughput thermal-shift assays. Methods. *Methods Molec. Biol.* **1729**, 281–290 (2018).

[32] Kranz, K. K. & Schalk-Hihi, C. Protein thermal shifts to identify low molecular weight fragments. *Methods Enzym.* **493**, 277–298 (2011).

[33] Wang, C. K., Weeratunga, S. K., Pacheco, C. M. & Hofmann, A. DMAN: a Java tool for analysis of multi-well differential scanning fluorimetry experiments. *Bioinf.* **28**, 439–440 (2012).

[34] Liu, X. *et al.* Distinct roles for conformational dynamics in protein-ligand interactions. *Structure* **24**, 2053–2066 (2016).
